# The Bile Acid Metabolism of Intestinal Microorganisms Mediates the Effect of Different Protein Sources on Muscle Protein Deposition in *Procambarus clarkii*

**DOI:** 10.3390/microorganisms13010011

**Published:** 2024-12-24

**Authors:** Xiaodi Xu, Xiaochuan Zheng, Qunlan Zhou, Cunxin Sun, Aimin Wang, Aimin Zhu, Yuanyuan Zhang, Bo Liu

**Affiliations:** 1 Wuxi Fisheries College, Nanjing Agricultural University, Wuxi 214128, China; 2021213005@stu.njau.edu.cn (X.X.); zhouql@ffrc.cn (Q.Z.); suncx@ffrc.cn (C.S.); 2 Key Laboratory for Genetic Breeding of Aquatic Animals and Aquaculture Biology, Freshwater Fisheries Research Center (FFRC), Chinese Academy of Fishery Sciences (CAFS), Wuxi 214081, China; 3 College of Marine and Biology Engineering, Yancheng Institute of Technology, Yancheng 224051, China; blueseawam@ycit.cn; 4 Yancheng Academy of Fishery Science, Yancheng 224051, China; zam--3@163.com; 5 Shandong Freshwater Fisheries Research Institute, Jinan 250013, China; yyuanzhang2008@163.com

**Keywords:** *Procambarus clarkii*, protein source, protein deposition, taurochenodeoxycholic acid, intestinal microbiota

## Abstract

The most economically important trait of the *Procambarus clarkii* is meat quality. Protein deposition is essential in muscle growth and nutritional quality formation. The effects and potential mechanisms of feed protein sources on crustaceans’ muscle protein deposition have not been elucidated. This study established an all-animal protein source (AP) and an all-plant protein source group (PP), with a feeding period of 8 weeks (four replicates per group, 45 individuals per replicate). The results demonstrated that muscle protein deposition, muscle fiber diameter, and hardness were significantly higher in the PP group (*p* < 0.05). The transcript levels of genes involved in protein synthesis were notably upregulated, while those of protein hydrolysis and negative regulators of myogenesis notably downregulated in PP group (*p* < 0.05). Furthermore, protein sources shaped differential intestinal microbiota composition and microbial metabolites profiles, as evidenced by a significant decrease in g_*Bacteroides* (*p* = 0.030), and a significant increase in taurochenodeoxycholic acid (TCDCA) in PP group (*p* = 0.027). A significant correlation was further established by Pearson correlation analysis between the g_*Bacteroides*, TCDCA, and genes involved in the *MSTN*-mediated protein deposition pathway (*p* < 0.05). In vitro anaerobic fermentation confirmed the ability of the two groups of intestinal flora to metabolically produce differential TCDCA (*p* = 0.038). Our results demonstrated that the ‘*Bacteroides*-TCDCA-*MSTN*’ axis may mediate the effects of different protein sources on muscle development and protein deposition in *P. clarkii*, which was anticipated to represent a novel target for the muscle quality modulation in crustaceans.

## 1. Introduction

*Procambarus clarkii*, also known as crayfish, is one of the most highly valued freshwater prawns in the aquaculture industry. The consumption demand for crayfish has increased in recent years, resulting in an expansion of both breeding areas and production. In 2023, China’s crayfish farming area was about 1.97 million hectares, producing 3.16 million tons [1]. The quality of crayfish is determined by its main edible part, i.e., the muscles, the most important economic trait that directly affects the market value of crayfish. As the market for crawfish expands and people’s living standards improve, consumers are demanding higher-quality prawn flesh. However, in the current crayfish aquaculture industry, large-size crayfish only constitute a low proportion, while most crayfish are undersized with muscles that are not full and firm enough. Therefore, how to comprehensively evaluate and improve the quality of crawfish muscle becomes a hot topic of industry attention.

Muscle quality mainly embodies physical and chemical characteristics (shearing force, water holding capacity, color, pH, etc.), nutritional quality (the deposition of crude protein and crude fat) and flavor quality (deposition of taste compounds). Nutritional quality is influenced by feed type and ingredients, particularly the protein source, which affects the turnover metabolism and deposition of protein in culture species [2]. Fish meal is a crucial protein source in aquafeeds. However, due to its limited supply and increasing price, feed costs have been increasing. Developing resourceful and inexpensive animal and plant protein sources that can become substitutes for fish meal according to native conditions, such as animal by-products of livestock and poultry, insects and cake meal, and intensively processed plant protein sources, is an important measure to overcome the bottleneck that hinders the development of aquaculture [3]. However, growth and health were identified as key indicators for screening new protein alternatives to fish meal [4]. There is a limited amount of research conducted from the perspective of muscle quality. Currently, studies only involve the effects on muscle quality characterization, with few studies reporting on the mechanism of actions [5,6]. The effects of plant and animal protein sources on muscle development and protein deposition in the crayfish remain poorly understood.

Previous studies have demonstrated that certain gut microbe-derived metabolites, including conjugated linoleic acid, acetate, and bile acids, can be transported to muscle tissues via the circulatory system [7]. Additionally, Laure et al. [8] have proved the existence of a ‘gut microbe–muscle axis’. In recent years, with the deeper excavation of microbial functions and the application of germ-free mice models [9] and tools such as fecal transplants [10] and antibiotic cleansing [11], it has been confirmed that intestinal flora are associated with the growth, development, and functional maintenance of muscles. The gut flora’s metabolites are important mediators in regulating host physiology. Limited reports have indicated that gut microbes produce specific secondary metabolites, including tryptophan [12], indolephenols sulfate [13], volatile fatty acids VFAs [14], bile acids BAs [15], and short-chain fatty acids SCFAs [16], which are involved in regulating myofibre development and muscle quality formation. Nevertheless, the investigation of the relationship between the intestinal microbes and their metabolites and muscle quality formation in aquatic animals is still in its preliminary stage, in contrast to the extensive studies conducted in human medical or mouse models. Wu et al. reported that gut microbial-derived butyric acid influenced muscle texture in Nile tilapia in the model of different protein sources feeding [17]. This finding suggests that the ‘gut–muscle axis’ may have potential applications in regulating fish muscle quality. However, the relationship between gut microbes, their derived metabolites, and muscle development and quality in crustaceans is currently unknown.

In light of the aforementioned considerations, the aim of this study is to investigate the effects of different protein sources on the muscle quality of crayfish, as well as to elucidate the roles and mechanisms of intestinal microorganisms and their derived metabolites in the protein deposition process in the muscle of crayfish. The present study offers a novel research perspective for the nutritional regulation of crustacean muscle quality under the hotly researched topic of alternative fish meal protein source development.

## 2. Materials and Methods

### 2.1. Ethics Statement and Experimental Design

The experimental procedures were conducted in accordance with the standards for scientific breeding and the utilization of crustaceans established by the Animal Care and Use Committee of the Committee on the Ethics of Animal Experiments of the Freshwater Fisheries Research Center (LAECFFRC-2023-04-28). All crayfish used in this trial were obtained from Jiangsu Jinfeng Agricultural Technology Co., Ltd. (Yancheng, China). After one week of acclimatization, a total of 360 crayfish with similar initial weight (4.92 ± 0.02 g) were randomly divided into two groups. Each of the four replicates in each treatment consisted of 45 individuals. The cement pond was used as the experimental setting (length × width × water depth, 2.5 m × 2.0 m × 0.4 m). In order to prevent crayfish from killing each other throughout the breeding period, a sufficient number of artificial nests, PVC pipes, and other shelters were placed in the cement ponds.

A total of the iso-nitrogen and iso-energy diets were designed in the feeding trial: All-animal protein sources group (AP group, whose diets comprise fish meal, chicken meal, hydrolyzed feather meal, pork meal, spray-dried blood cell powder, and shrimp powder); All-plant protein sources group (PP group, whose diets comprise soybean meal, rapeseed meal, peanut meal, rice protein concentrate, soybean protein concentrate, and DDGS); The formulations and approximate compositions of the experimental diets are shown in Table 1.

In this experiment, the proximate analysis of the feed is conducted in accordance with the guidelines of the AOAC (2016) standard [18]. The crude protein level of the feed was measured by Kjeldahl nitrogen determination (FOSS KT260, Zurich, Switzerland); the ether extract content of the feed was determined through Soxhlet extraction (Tecator, Hoganas, Sweden); the gross energy was analyzed using an oxygen bomb calorimeter (Parr, Moline, IL, USA); heat the feed until it was smoke-free and carbonized, then burn it at 550 ± 25 °C for 4 h using a muffle furnace. After the temperature dropped to 200 °C, the feed was dried for 30 min to calculate the ash content.

### 2.2. Crayfish Feeding, Growth Assay and Sampling

Crayfish were fed twice each day at 6:00 a.m. and 20:00 p.m. with a 5–10% feeding rate for 8 weeks. The feeding rate was adjusted according to the amount of residual bait. The rearing conditions were maintained at a temperature of 28–32 °C, a pH range of 7.0–7.5, and dissolved oxygen levels above 5 mg/L, with ammonia level below 0.1 mg/L. Mortality rates were recorded daily. After 8 weeks of feeding, the weight gain rate (WGR), specific growth rate (SGR), hepatopancreatic index (HSI), and meat rate were analyzed as the following formulas:

$$WGR\;(\%)=\frac{G2-G1}{G1}\times 100\%;$$

$$SGR\;(\%)=\frac{Ln\;G2-Ln\;G1}{days}\times 100\%;$$

$$FCR=\frac{G3}{G4}$$

$$HSI\;(\%)=\frac{G5}{G2}\times 100\%;$$

$$Meat\;rate\;(\%)=\frac{G6}{G2}\times 100\%.$$

Notes: G1 is initial weight (g), G2 is final weight (g), G3 is dry feed intake (g), G4 is weight gain (g), G5 is hepatopancreas weight (g), G6 is meat weight (g).

Some fresh muscle samples taken from the same muscle segment position were collected for muscle texture analysis (eight prawns per cement pond), nutritional composition determination (three prawns per cement pond), and histology and ultrastructural observation (one prawn per cement pond). The remaining sufficient muscle tissue were stored at −80 °C for subsequent molecular analysis. Sufficient intestinal tissue and contents were also collected aseptically and stored at −80 °C for the determination of microbiological indicators.

### 2.3. Muscle Quality Analysis

One piece of dorsal muscle per prawn was cut to a uniform size (8 mm × 8 mm × 6 mm) for analysis of muscle texture parameters using a TA-XT2i texture analysis instrument (Stable Micro Systems Ltd., Godalming, Surrey, UK). The texture parameters of hardness, springiness, cohesiveness, gumminess, chewiness, and resilience were analyzed using a cylindrical stainless-steel plunger with a diameter of 50 mm (P/50). The probe exhibited a pre-test speed of 1 mm/s, a test speed of 1 mm/s, a post-test speed of 5 mm/s, a trigger force of 20 g, and a compression equivalent to 50% of the meat thickness. The texture parameter of shearing force was measured using a A-MORS probe, with a pre-test speed of 1.5 mm/s, a test speed of 1.5 mm/s, a post-test speed of 10 mm/s, and a trigger force of 20 g. The TPA parameters were then calculated using software provided in the User Guide (Version 1.0).

A portion of the muscle samples were subjected to the proximate analysis in accordance with the guidelines of the AOAC (2016) standard. Amino acid composition was determined for each prawn sample using an automatic amino acid analyzer (Gel Chromatograph, LC98IIRI, Beijing Wenfen Analytical Instrument Development Co., Ltd., Beijing, China), with 30 mg of muscle tissue being used for each analysis [19].

### 2.4. Morphological Analysis of Muscle Tissue

From each replicate, a small portion of muscle tissue was excised and fixed with a glutaraldehyde solution for 24 h and then with 1% osmium acid for 1 h. The fixed samples were then dehydrated in acetone, embedded in Epon812 epoxy resin, sliced ultrathin (70 nm), and stained with uranium acetate-lead citrate. The sarcomere length was measured using a transmission electron microscope (Hitachi H-7650, Tokyo, Japan). The remaining muscle tissue was fixed with 4% paraformaldehyde buffer over a period of 24 h. The fixed samples were then dehydrated using a gradient concentration ethanol series (30% for 2 h, 50% for 1 h, 75% for 80 min, 85% for 40 min, 95% for 30 min, and 100% for 30 min, then 100% for another 30 min). The samples were then transparent sliced using a 1:1 mixture of alcohol and xylene for 30 min, xylene for 30 min, and xylene for 30 min again. The samples were then embedded with paraffin wax and sliced to 5 μm (Leica RM2255, Nussloch, Germany). The slices were then dewaxed, hydrated, and finally stained with hematoxylin and eosin (H.E.) [20]. Five different fields were randomly selected using a microscope (50×, Olympus BX53; Olympus, Tokyo, Japan) equipped with a digital camera (Olympus DP73; Olympus, Tokyo, Japan), and the area was calculated by fitting the muscle fiber to a circle using the Image J software (version 1.53e, National Institutes of Health, Bethesda, MD, USA), and the muscle fiber diameter (μm) was derived using the formula (S = πr^2^, d = 2r).

### 2.5. Determination of Muscle Molecular Indicators

The detailed procedures including RNA extraction and integrity determination, cDNA synthesis, and qRT-PCR were carried out in accordance with the protocols described by our previous report. The *EIF* gene was designated as the reference gene, with each sample being subjected to triplicate tests in order to obtain accurate results. Primer design was carried out using online design tools (available from NCBI, Bethesda, MA, USA) (Table 2). Several primers were designed for selected genes using the CDS sequences of muscle transcriptome sequencing of *P. clarkii* from the laboratory’s database. The PCR primers being synthesized by Shanghai Sangon Biotechnology, Co., Ltd., Shanghai, China. The resulting data were analyzed using the 2^−ΔΔCt^ method.

### 2.6. 16S rDNA Sequencing and Metabolomics Analysis of Chyme

Aseptic collection of the crayfish chyme was undertaken for the purpose of 16S rDNA sequencing. The DNA extraction and concentration measurement were conducted using the E.Z.N.A.^®^ Soil DNA Kit (Omega Bio-Tek, Norcross, GA, USA) and the Quant iT PicoGreen dsDNA Assay Kit (Invitrogen, Carlsbad, CA, USA), respectively. The processes of PCR amplification, product target fragment collection, purification, and quantification are described in details in our previous study [26]. The library construction and double-ended sequencing were conducted using the Illumina HiSeq platform (Illumina, Inc., San Diego, CA, USA), and the raw fastq files were obtained after the removal of the barcode and linker sequences. The paired-end sequences were assembled, deduplicated, denoised, and clustered to generate OTUS using the FLASH software (version 1.2.8, http://ccb.jhu.edu/software/FLASH), the VSEARCH software (https://github.com/torognes/vsearch, version 2.3.4), and the UPARSE software (version 7.1), respectively.

The additional chyme was collected from the AP and PP groups in an aseptic manner. The supernatant was prepared by thorough grinding with pre-cooled methanol-water (1:1, *v*/*v*), followed by centrifugation (4 °C, 2500× *g*) and redissolution with methanol-water (1:9, *v*/*v*), and finally, ultrasonication. Subsequently, an LC-MS/MS analysis was performed utilizing an ACQUITY UPLC BEH C18 column (100 mm × 2.1 mm, 1.7 µm, Waters, Cheshire, UK) at a temperature of 50 °C. The high-resolution tandem mass spectrometry Xevo G2-XS QTOF (Waters Corporation, Milford, MA, USA) was employed to obtain the raw data. Following the removal of low-quality ions, the differential metabolites were identified through multivariate analysis of variable importance in projection (VIP) values derived from the first two principal components of the partial least squares discriminant analysis (PLS-DA) model, in conjunction with univariate analysis of fold change and *p*-value. For detailed steps and parameters of metabolomics, refer to our previous description [27].

### 2.7. In Vitro Anaerobic Fermentation of Chyme and BAs File Analysis

Approximately 1 g intestinal contents were aseptically dissociated from AP and PP groups. Firstly, homogenize the chyme sample to prepare a 10% (*w*/*v*, 1:9, 0.1 mol/L 10 × PBS solution) fecal bacterial suspension, which was then treated with a high-temperature and high-pressure sterilization. The components of the culture medium are as follows: dipotassium hydrogen phosphate 0.4 g/L, potassium dihydrogen phosphate 0.4 g/L, peptone 2 g/L, yeast extract powder 2 g/L, sodium bicarbonate 2 g/L, sodium chloride 0.1 g/L, magnesium sulfate heptahydrate 0.1 g/L, calcium chloride 0.1 g/L, T802 mL/L, cysteine hydrochloride 0.5 g/L, bile salt 0.5 g/L, vitamin K1 solution 10 μL/L, heme chloride 0.05 g/L, and sodium thioglycolate 3 g/L. After sterilization and sterile filtration, the vitamin K1 solution, hemin solution, deoxygenator C_2_H_3_NaO_2_S, and fecal bacterial solution were added into the medium. Anaerobic fermentation was carried out in a 15 mL anaerobic glass tube, and a 6 mL sample was cultured at 30 °C for 12 h in the incubator. Subsequently, 1 mL of the sample underwent centrifugation at 4 °C, 12,000× *g* for 10 min, and the resultant supernatant was used for quantitative analysis of BAs. The types and concentrations of BAs in the fermentation broth were determined by subjecting the samples to LC-MS analysis using a Waters ACQUITY UPLC system coupled with a Waters XEVO TQ-S Mass Spectrometer (Waters Corporation, Milford, MA, USA). LC-MS/MS analysis was performed using a Waters Acquity BEH C18 column (100 mm × 2.1 mm, 1.7 μm) with a mobile phase consisting of a mixture of 0.1% formic acid in water and 0.1% formic acid in acetonitrile (temperature, 45 °C; flow rate, 0.4 mL/min). Subsequent to gradient chromatographic elution, mass spectrometry detection and calibration equations matching identification were conducted in negative mode using the Waters TargetLynx Application Manager (version 4.1, Waters Corporation, Milford, MA, USA). For detailed steps and parameters of metabolomics, refer to our previous description [26].

### 2.8. Statistical Analysis

The data were analyzed by an independent sample *t*-test using SPSS 20.0 software. The results are expressed as means ± S.E.M. *p* < 0.05 was considered to indicate a significant difference. Prior to the analysis, all data were tested for normal distribution and variance homogeneity using Levene’s test. Correlation analyses were conducted using Pearson’s correlation test.

## 3. Results

### 3.1. Growth Performance and Proximate Analysis of Muscle

Table 3 showed an increase in FW (13.10%), WGR (15.84%), and SGR (6.66%) levels of PP group, while in contrast, those of AP group have no significant differences (*p* > 0.05). The FCR decreased significantly (14.70%, *p* = 0.011), while the crude protein level increased significantly in the PP group in comparison to these of AP group (7.04%, *p* = 0.030).

### 3.2. Amino Acid Composition in the Muscle

**Figure 1:**
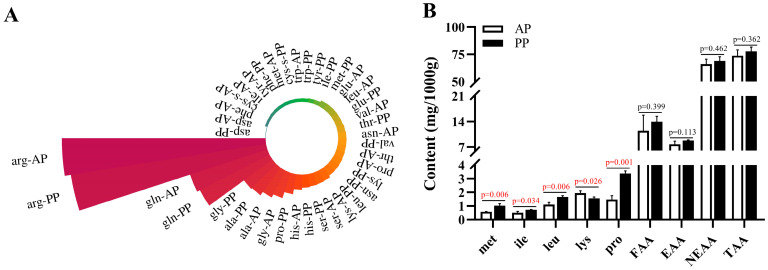
The effects of animal and plant protein sources on the amino acid composition in the muscle of *P. clarkii*. Note: (**A**) Amino acid composition in the AP and PP groups; (**B**) Differentiated amino acids and classification; AP, all-animal protein source group; PP, all-plant protein source group. Red data mean the *p* value < 0.05.

As illustrated in Figure 1A,B, the contents of methionine, isoleucine, leucine, and proline in PP group were significantly increased in comparison to AP group (*p* < 0.05), while lys in PP group was significantly decreased than that of AP group (*p* = 0.026). No statistically significant difference was found between these two groups in the indicators of ∑EAA, ∑NEAA and TAA (*p* > 0.05).

### 3.3. Muscle Histological Morphology

**Figure 2:**
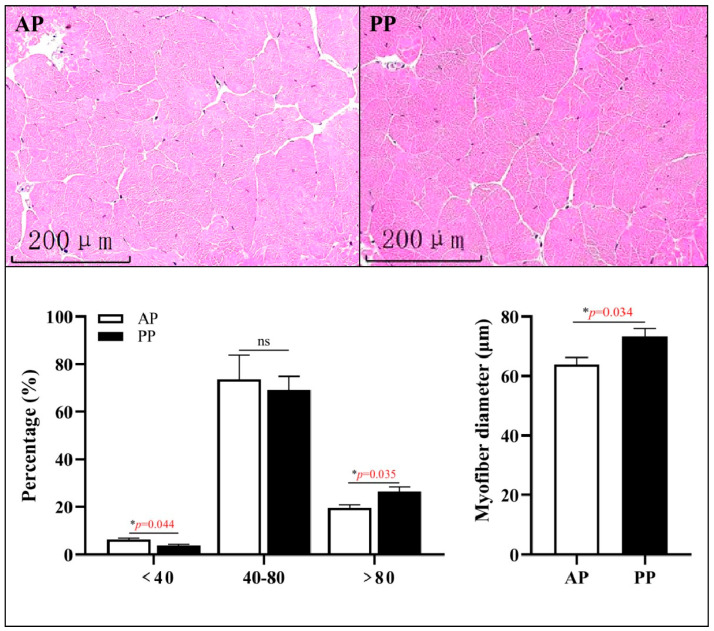
The effects of animal and plant protein sources on the histological morphology of muscle in *P. clarkii*. Note: AP, all-animal protein source group; PP, all-plant protein source group. Red data with asterisk mean the *p* value < 0.05, and ns means no significant difference.

Figure 2 showed that the PP group exhibited a notable elevation in the mean myofiber diameter compared with the AP group (*p* = 0.034), especially the ratio of fibers with a diameter greater than 80 μm exhibited a pronounced increase in the PP group (*p* = 0.035).

### 3.4. Muscle Texture Analysis

**Figure 3:**
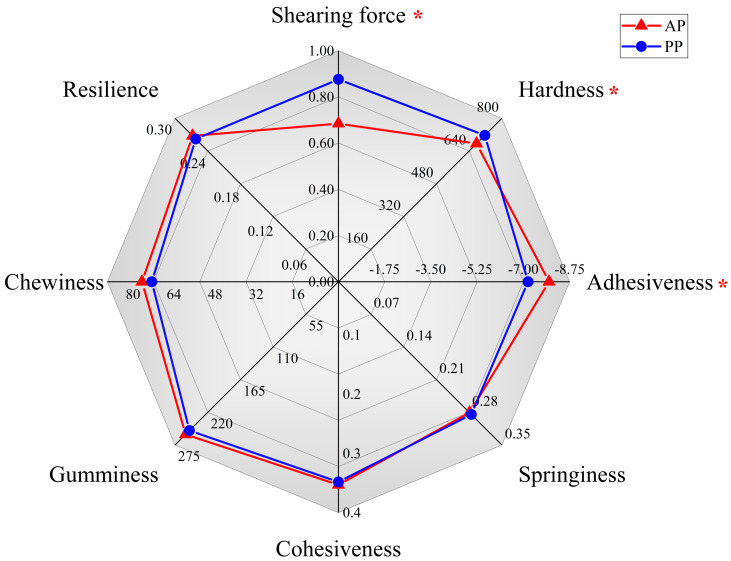
The effects of animal and plant protein sources on the muscle texture characteristics of *P. clarkii.* Note: Red means the texture parameters of AP group, blue means the texture parameters of PP group. * means *p* < 0.05. AP, all-animal protein source group; PP, all-plant protein source group.

As illustrated in Figure 3, the shearing force and hardness were significantly increased while the adhesiveness was significantly decreased in the PP group than those of AP group (*p* < 0.05). No significant difference was found between these two groups in the indicators of springiness, cohesiveness, gumminess, chewiness, and resilience (*p* > 0.05).

### 3.5. Muscle Development Related Genes Expression

**Figure 4:**
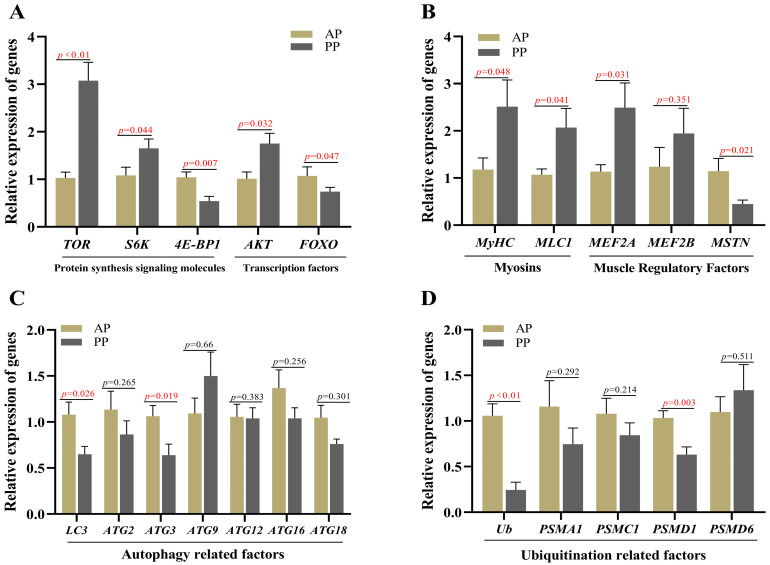
Effects of animal and plant protein sources on the transcription levels of genes related to muscle development in *P. clarkii*. Note: (**A**) Relative expression of genes related to protein synthesis signaling molecules and transcription factors; (**B**) Relative expression of genes related to myosins and muscle regulatory factors; (**C**) Relative expression of genes related to autophagy factors; (**D**) Relative expression of genes related to ubiquitination factors. AP, all-animal protein source group; PP, all-plant protein source group; Red data means the *p* value < 0.05.

RT-PCR was performed to detect muscle development related gene expression, involving protein synthesis, myogenic factors, autophagy, and ubiquitination. The results are presented in Figure 4. The PP group had significantly upregulated muscle protein synthesis regulators (*AKT*, *mTOR*, *S6K1*), myosin and myogenesis regulators (*MEF2A*, *MEFAB*, *MyHC*, *MLC1*), and significantly downregulated (*p* < 0.05) *MSTN*, muscle protein hydrolysis-autophagy, and ubiquitination-related factors (*FOXO*, *LC3*, *ATG3*, *Ub*, *PSMD1*) (*p* < 0.05).

### 3.6. 16S rDNA Analysis and Metabolites Profile of the Intestinal Chyme

**Figure 5:**
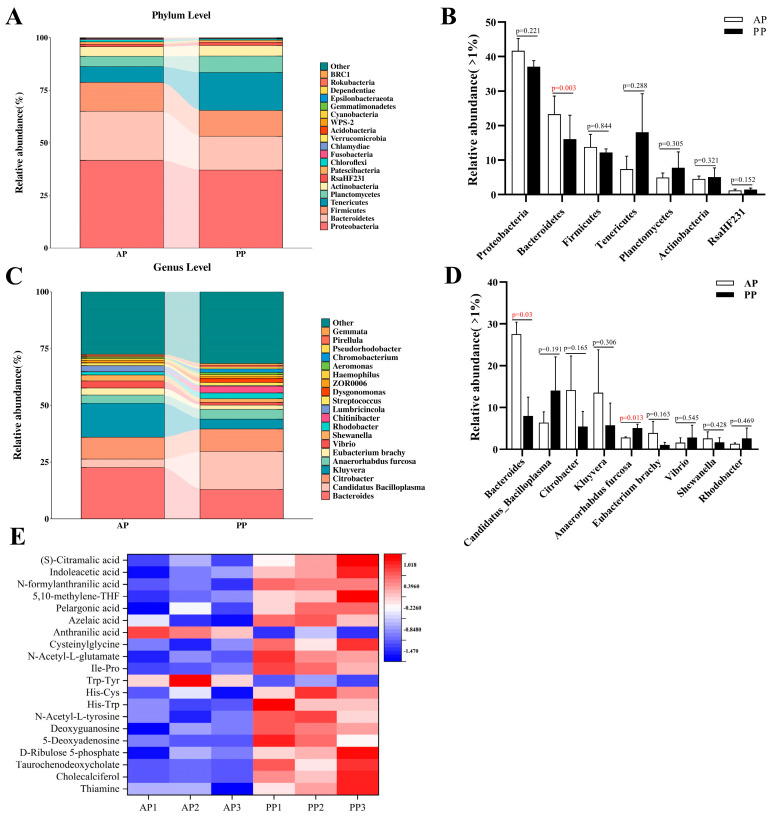
Effects of animal and plant protein sources on the intestinal microbiota structure and intestinal content composition of *P. clarkii*. Note: (**A**,**B**), The relative abundance of the top seven predominant phyla (with a mean relative abundance > 1%). (**C**,**D**), The relative abundance of the top seven predominant phyla (with a mean relative abundance > 0.01%). (**E**), Heatmap analysis of 20 differential metabolites. AP, all-animal protein source group; PP, all-plant protein source group. Red data means the *p* value < 0.05.

The structure of the intestinal flora of crayfish was compared using 16S rDNA sequencing after different protein sources treatment (Figure 5A–D). At the level of the phylum, the abundance of Bacteroidetes was significantly reduced in the PP group compared to that of AP group. With regard to the genus level, the PP group showed a significant downregulation in g_*Bacteroides* abundance and a significant upregulation in the abundance of g_*Anaerorhabdus furcosa* compared to the AP group. Metabolomics was conducted to comparatively analyzed the effects of protein sources on the metabolites composition of crayfish intestinal contents (Figure 5E). Eighteen metabolites, including TCDCA and cholecalciferol, were significant higher in the PP group, while anthranilic acid and Trp-Tyr were significantly downregulated compared to the AP group.

### 3.7. Correlation Analysis

**Figure 6:**
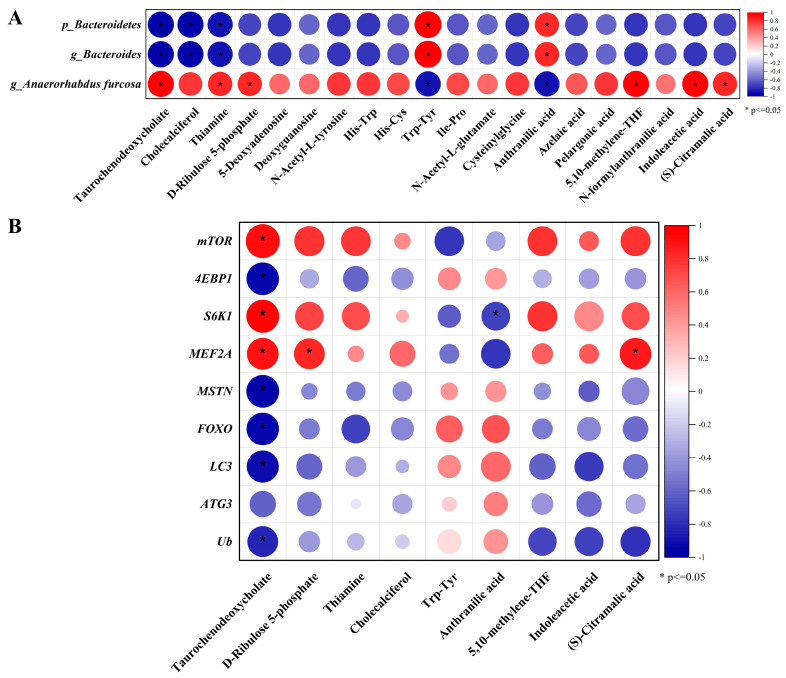
Correlation analysis. Note: (**A**) Intestinal microbiome and differential metabolites correlation analysis; (**B**) Correlation analysis of selected differential metabolites and muscle development-related genes. Red ball means positive correlation, while blue ball means negative correlation, * means *p* < 0.05.

Correlations between differential intestinal microbes, differential chyme metabolites, and muscle development-related differential genes were evaluated using Pearson’s correlation analysis. Among them, Bacteroidetes and g_*Bacteroides* had a significant negative correlation with TCDCA, Cholecalciferol, and Thiamine. Moreover, Bacteroidetes and g_*Bacteroides* had a significant positive correlation with Trp-Tyr and anthranilic acid (Figure 6A). g_*Anaerorhabdus furcosa* was significantly positively correlated with TCDCA, Thiamine, D-Ribulose 5-phosphate, 5,10-methylene-THF, Indoleacetic acid, (s)-Citramalic acid, and had a significant passive correlation with Trp-Tyr, and anthranilic acid (Figure 6A). Further correlation analyses of metabolites with muscle development-related differential genes showed that TCDCA was significantly positively correlated with *mTOR*, *S6K1*, and *MEF2A*, and had a significant negative correlation with *MSTN*, *4EBP1*, *FOXO*, *LC3*, and *Ub*; D-Ribulose 5-phosphate had a significant positive correlation with *MEF2A*; Anthranilic acid had a significant negative correlation with *S6K1*; (s)-Citramalic acid had a significant positive correlation with *MEF2A* (Figure 6B).

### 3.8. Bile Acid Profile Quantity of In Vitro Intestinal Anaerobic Fermentation Broth

**Figure 7:**
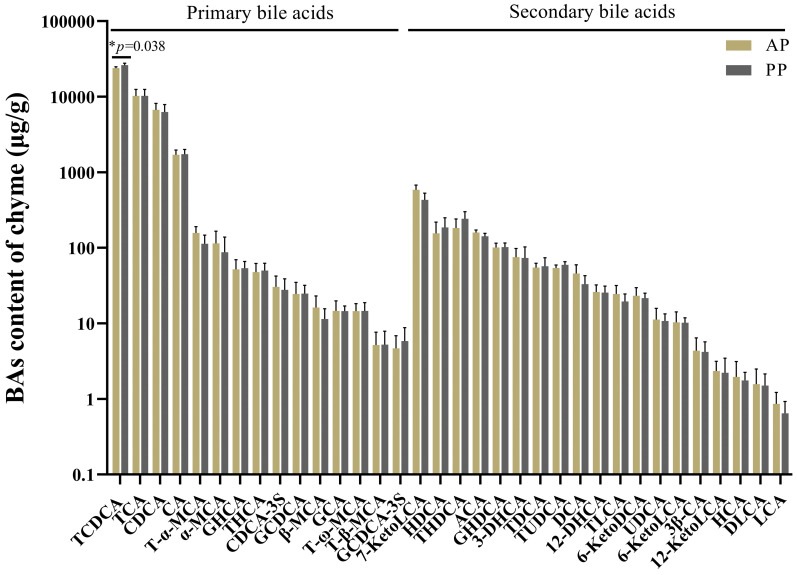
Bile acid profile of in vitro intestinal anaerobic fermentation broth. Note: Independent Samples *t*-test was used to detect the significant differences between the AP and PP group. * means *p* < 0.05. AP, all-animal protein source group; PP, all-plant protein source group.

As presented in Figure 7, the intestinal chyme of the *P. clarkii* in the AP and PP groups were subjected to in vitro anaerobic fermentation and the variations in bile acid profiles were measured. In total, 34 bile acids were identified through targeted metabolomics analysis, including 15 primary bile acids and 19 secondary bile acids. TCDCA is the metabolite with the highest content, and the TCDCA content in PP group was significantly increased in comparison to the AP group (*p* = 0.038). Other bile acids were not significantly different between groups (*p* > 0.05).

## 4. Discussion

The current investigation on the effects of different protein sources on the muscle development and quality in aquatic animals is limited. This study aims to evaluate the effect of different protein sources on the meat quality of crayfish. There was no significant difference in growth performance of crayfish consuming two different types of protein sources. Meanwhile, a significantly higher muscle crude protein deposition was found in the all-plant protein group. One similar study reported that the efficiency of protein synthesis as well as protein deposition were significantly higher in diet with sunflower meal as the protein source than the diet with meat powder as the sole protein source in the *Anguilla anguilla* [28]. Ibrahim et al. found that kidney bean protein hydrolysate could replace up to 100% of the fish meal, with improvements in growth and associated genes expression and suppression in myostatin gene expression [29]. Also, other studies have found that the growth and protein retention of Atlantic cod [30] and Atlantic salmon [31] were not negatively affected by replacing the 100% fishmeal with plant proteins. It demonstrated that the plant component was of high quality, the AA composition of the diet was in a state of well equilibrium, and the feed intake was comparable to that of the fishmeal control diet. The above studies indicated that the quality of feed protein ingredients affected protein deposition in aquatic animals. One recent study proposed that the reason why different protein sources affected the biosynthesis and degradation metabolism of muscle protein was mainly achieved by the balance of amino acid composition [5]. Amino acid imbalances are linked to reduced protein synthesis or enhanced protein breakdown, or to both components of protein turnover being altered at the same time. In this study, we noticed that the methionine, proline, and branched-chain amino acids leucine and isoleucine contents in muscle tissue were significantly higher in the PP group compared to those in the AP group. Branched-chain amino acids, especially leucine, could improve protein synthesis efficiency [32]. Methyl donor methionine contributed to protein turnover and protein synthesis in skeletal muscle [33]. The proline could promote collagen protein synthesis in muscle [34]. The muscle AA composition analysis results demonstrated that the amino acid profile and balance of the plant protein diet, comprising soybean meal, rapeseed meal, peanut meal, rice protein concentrate, soybean protein concentrate, and DDGS, were superior to those of the all-animal protein source diet, which consisted of fishmeal, chicken meal, feather meal, blood cell powder, and dried shrimp shell powder. The preceding results demonstrated that we could not simply define how good or bad a particular protein source was. Rather, the overall nutritional composition of the ration, namely the composition and balance of AAs and FAs, is the essence that determines the nutritional value of the feed.

Mouthfeel is a vital evaluation index of meat quality, which can be reflected by hardness and shear force. In terms of shrimp, higher hardness and elasticity were regarded as better mouthfeel of shrimp flesh. Research confirmed that muscle hardness positively correlated with collagen content [35] and fiber histological characteristics (diameter and density) [17,36], and the shear force positively correlated with myofiber diameter and area [37]. In this study, the muscle textural parameters, such as shear force and hardness, of all-plant protein group was higher than all-animal protein group. Consistent with this, higher muscle collagen precursor proline and muscle fiber diameter and density were found in the all-plant protein group. Similarly, studies confirmed that different protein sources affect the muscle texture parameters and physical properties of muscle [6,38]. The fundamental process underlying muscle fiber growth is protein deposition. Muscle fiber hypertrophy correlates closely with the amount of protein deposition. The balance between protein synthesis and catabolism controls muscle protein deposition and muscle mass. Protein synthesis is primarily regulated by mTOR signaling. mTOR regulates muscle protein synthesis by phosphorylation activation of S6K1 kinase and phosphorylation inactivation of 4EBP1, and inhibition of mTOR signaling leads to decreased muscle function and muscle loss [39]. Protein degradation is mainly regulated through two pathways: the ubiquitin-proteasome and autophagy lysosome pathways [40]. In this study, the prawns fed with all-plant protein diet showed significant higher transcription level of muscle protein synthesis regulators (*AKT*, *mTOR*, *S6K1*), myocyte enhancer factors (*MEF2A*, *MEF2B*), and myogenesis regulators (*MyHC*, *MLC1*), and significant lower transcription levels of muscle protein hydrolysis-autophagy and ubiquitination-related factors (*FOXO*, *LC3*, *ATG3*, *Ub*, *PSMD1*). Among them, FoxOs were crucial transcription factors that regulate both the ubiquitin and autophagy pathways [41]. The aforementioned results suggested that different protein sources influenced the muscle protein deposited by affecting the equilibrium between protein synthesis and degradation. It is noteworthy that the muscle growth inhibitor (myostatin) *MSTN* regulates the dynamic equilibrium of muscle protein synthesis and catabolism by targeting AKT pathway in vertebrates. This is exemplified by protein synthesis through the AKT/mTOR pathway and protein catabolism through the AKT/FOXO-mediated ubiquitin-protein hydrolase system and autophagy lysosome pathway [42,43]. MSTN is also implicated in many signaling pathways controlling skeletal muscle growth and development. The interaction of MSTN with the SMADS family of proteins negatively regulates the expression of the MRFs family of proteins (Myf5, MyoD and MyoG), thereby inhibiting myoblast proliferation, differentiation, and growth. It can therefore be concluded that MSTN is a negative regulator of protein deposition and myofiber development in vertebrates. In invertebrates, MSTN appears to have a similar function in muscular development with mammalian MSTN, such as *Fenneropenaeus chinensis* [44], *Exopalaemon carinicauda* [45], and *Macrobrachium rosenbergii* [46]. In this study, significantly lower MSTN and higher myocyte growth indicators were found simultaneously in PP group than those in AP group, indicating that PP diet reversed the negative regulation of MSTN to promote muscle protein deposition and muscle development of *P. clarkii*. However, unlike mammals, MSTN seems to be associated with more physiological functions in invertebrates, such as molting [47]. Further research is needed to clarify its comprehensive functions.

It has been shown that modification of the gut microbiota by bacterial depletion, fecal transplantation and nutritional supplementation has a direct impact on muscle phenotypes, confirming the existence of a gut-muscle axis in vertebrates [48]. Cues (microbial products) from the gut, through its interaction with the gut microbiome, provide a contact between the gut microbiome and musculature [49,50]. The above foundation makes us think: does the intestinal microbiota and its derived metabolites also participate in the regulation of muscle fiber development and flesh quality formation in crustaceans? What is the specific pathway of the gut-muscle axis in crustaceans? Consequently, we further investigated the response of gut microbes and their metabolites to different protein sources, and their potential roles in the muscle development and deposition of proteins in crayfish. In our study, the correlation between the differentiated intestinal bacterial genus structure (*Bacteroides*), bile acid metabolite (taurodeoxycholic acid, TCDCA), and genes related to the *AKT*/*mTOR*/*FOXO* protein synthesis/degradation pathway mediated by MSTN was confirmed based on microbiome, gut content metabolome, and Pearson’s correlation analysis. Previously, there was considerable doubt as to whether a bile acid metabolic pathway similar to that of vertebrates existed in crustaceans. This was because information on bile acid synthesis genes and receptor genes had not been reported, and the effects and mechanisms of crustacean gut microorganisms on bile acid metabolism were also unclear in crustaceans. However, with the advent of metabolomics technology, researchers have recently identified the existence of distinct bile acid profiles in crustaceans, analogous to those observed in vertebrates, including deconjugated primary bile acids, conjugated primary BAs, and secondary BAs. Examples of crustaceans exhibiting these profiles include the *Macrobrachium rosenbergii* [26] and the *E. sinensis* [34]. In vertebrates, the liver metabolizes cholesterol to synthesize primary bile acids, which are conjugated with taurine or glycine in the blood and are metabolically modified to secondary bile acids by specific genera of gut microorganisms, such as *Bacteroides*, *Clostridium*, *Lactobacillus*, etc. Therefore, differentiated intestinal flora structures and bile acid metabolizing enzyme activities of the flora result in differentiated bile acid profiles. TCDCA is a conjugated primary bile acid, the significantly upregulated TCDCA in the PP group may be attributed to the significantly decreased *Bacteroides*, catalyzing the hydrolysis of glycine and/or taurine bound bile salts to amino acid residues and deconjugated BAs with BSH enzymes [51]. In accordance with this, in vitro anaerobic fermentation of intestinal contents demonstrated that TCDCA was the most abundant bile acid in crayfish, with significantly higher levels observed in the PP group. This result corroborates the capacity of differential composition of intestinal microbiota to produce differential TCDCA abundance between these two groups. TCDCA has been the subject of greater investigation with regard to its anti-inflammatory and immunomodulatory functions through the TGR5 receptor [52,53]. Recent studies in mammals have demonstrated that the activation of the expression of two classical receptors for bile acids, TGR5 and FXR, in muscle tissues is closely related to muscular dystrophy and muscle hypertrophy [54]. These reports indicate that the relationship between bile acids and skeletal muscle development may be closer than previously thought. One report indicated that bile acids could promote breast muscle growth in chickens through the FXR/IGF2 pathway [36]. Treatment with deoxycholic acid (DCA) and cholic acid (CA) resulted in a reduction in myofiber diameter and MyHC protein levels, accompanied by an increase in the expression of muscle fiber atrophy-associated proteins, atrogin-1 and MuRF-1, in mice. TGR5 knockdown resulted in the complete elimination of the aforementioned effects induced by DCA and CA, as well as the over-activation of the protein hydrolysis system (ubiquitin-proteasome system) [55]. UDCA induces sarcopenia in mice, presenting as decreased protein synthesis and altered autophagic flux, and sarcopenic-like features in C2C12 myotubes and/or isolated muscle fibers [56]. Lithocholic acid (LCA) has the capacity to activate the TGR5/AKT pathway, inhibiting protein degradation, promoting protein synthesis and enhancing the myogenic process [57]. The aforementioned findings indicated that distinct types of bile acids might exert varying influences on muscle growth and development, as well as protein deposition.

In conclusion, our study revealed that different protein sources had varying effects on muscle protein deposition and muscle fiber development, and the “*Bacteroides*-TCDCA-*MSTN*” pathway participated in this physiological process. However, this study has certain limitations. Firstly, it is necessary to ascertain whether intestinal microbiota exerts an influence on muscle physiology using intestinal microbiota transplantation technology. Furthermore, feeding validation studies are required to evaluate the regulatory effects of candidate metabolite TCDCA on the muscle protein deposition and muscle fiber development in *P. clarkii*. The findings of the present study offer a novel perspective and theoretical foundation for the nutritional regulation of muscle quality targeting “gut microbes–muscle axis” in crustaceans.

## Figures and Tables

**Table 1:** Components and proximate analysis of AP diets and PP diets.

	AP	PP
Components (% dry matter)		
Fish meal	17.0	0.0
Chicken meal	15.0	0.0
Hydrolyzed feather meal	3.0	0.0
Pork powder	3.0	0.0
Spray-dried blood cell powder	5.0	0.0
Shrimp meal	5.0	0.0
Soybean meal	0.0	25.0
Rapeseed meal	0.0	18.0
Rice protein concentrate	0.0	8.0
Soybean protein concentrate	0.0	5.0
Peanut meal	0.0	5.0
DDGS	0.0	3.0
Rice bran	4.0	4.0
Salt	0.3	0.3
Starch	36.99	18.79
Soybean oil	0.8	3.0
Squid paste	3.0	3.0
Ecdysone (2%)	0.01	0.01
Vitamin premix ^a^	1.0	1.0
Mineral premix ^a^	1.0	1.0
Choline chloride (50%)	0.5	0.5
Calcium dihydrogen phosphate	2.0	2.0
Carboxymethyl cellulose	0.5	0.5
Bicarbonate	1.5	1.5
Microcrystalline methionine	0.4	0.4
Total	100.0	100.0
Proximate analysis (%)		
DM	83.49	83.27
CP	33.29	33.21
EE	5.91	6.08
Gross energy (MJ/kg)	16.69	16.09

Note: ^a^ Premix obtained from Wuxi Tongwei Feed Co., Ltd., Wuxi, China.

**Table 2:** Primer sequences for real-time PCR.

Gene	Forward (5′-3′)	Reverse (5′-3′)	PL (bp)	Reference
House keeping gene				
*EIF*	GGAATAAGGGGACGAAGACC	GCAAACACACGCTGGGAT	126	[21]
Protein synthesis signaling molecules				
*TOR*	GAAGGCATGCTGCGGTATTG	CGCAGGCTTTGGGTCTCTTA	122	[21]
*S6K*	ACAGCCGAGAATCGCAAGAA	ATCACCATTATCGGGTCCGC	153	[21]
*4E-BP1*	ACCTGCCAGTGATACCAGGA	TGGCTCCTCTGAAATCGTTCC	80	[21]
*AKT*	CCTTGGGGCGTCTACTCCTA	TCCTCATAATCCTCACTTTCCT	176	database
Muscle regulatory factors				
*MyHC*	AAGCCAACCGTACCCTCAA	AGTAGCACGTTCTCTGCATTCA	174	database
*MLC1*	TGAGAAGGTCGGAGGCAAG	TGCCATTCTCAGATTTGTCGT	155	[22]
*MEF2A*	CATCTTCCAACCATCCTGGG	GTTTGCTCAACGGGGTATCA	125	[22]
*MEF2B*	ACCAGCACCACCTTCACATT	GAAGATGGACCCAAATGTGAA	133	[22]
*MSTN*	AGCAACAGCAACAACAAGGA	GCAGGAAGGGACATTTACCG	136	[22]
Autophagy related factors				
*FOXO*	ACGCGCTAACACCATGGAAG	GACTCTCACTCAGCGACGAA	158	[23]
*LC3*	TGAGTAGTCCGTCTCGGTGT	CCATGTAGAGGAACCCGTCG	169	[24]
*ATG2*	GTACTTCCCGTGGTCGGATG	CCATCCACGAACCTGAGAGG	175	[24]
*ATG3*	GCCAAGACAACCACCATAGC	AGAGCCGAGGTGTCTGGTAG	201	database
*ATG9*	TCATACATCCAGGGTTCGCC	GGGCAAAGGAACAAACGTCC	189	database
*ATG12*	TGGAGGGGAAGGACTTACGG	AGCTTTCCCTTAGCAGTCTTC	203	[24]
*ATG16*	AGATGGATGGCACAGAAGGC	GTTCACTTGCTTGGGCTCAC	178	database
*ATG18*	CGTGTTGTAGTGGAGGAGCA	CGTGGCTGCTTTTGAATCGT	194	database
Ubiquitination related factors				
*ub*	TCCAGCCTCTCCTGCCTT	CCTTCCTTATCCTGAATCTTTGCC	172	[25]
*Psma1*	CTTTACCTCATTGACCCATCT	CACAACCATAGTATCCATTACACAT	149	database
*psmd1*	ACTCATACAGCAAACAGAATCC	CGTCCACCAGCATCAATAA	147	database
*psmd6*	AGCTTTTGCTAAAACCTACG	TCCCAATCTCCTCCCTCT	159	database
*Psmc1*	TGTCTCCATTCTCTCCTTTGT	TTGAGGTGCCTTCTCTAGCT	148	database

**Table 3:** The effects of animal and plant protein sources on the growth performance and muscle nutritional composition of *P. clarkii*.

Indicators	AP	PP	*p*-Value
IW (g)	4.93 ± 0.014	4.92 ± 0.028	0.833
FW (g)	30.84 ± 0.295	34.88 ± 1.389	0.059
WGR (%)	525.91 ± 7.130	609.23 ± 30.671	0.069
SGR (%/day)	3.53 ± 0.022	3.77 ± 0.084	0.064
SR (%)	89.17 ± 2.52	90.04 ± 2.00	0.766
FI (g)	22.57 ± 0.932	22.26 ± 0.832	0.807
FCR	0.87 ± 0.026	0.74 ± 0.025	0.011
HSI (%)	7.32 ± 0.236	6.74 ± 0.213	0.071
Meat rate (%)	10.13 ± 0.434	11.62 ± 0.905	0.098
Proximate analysis of muscle (%)			
Moisture content	76.16 ± 0.302	75.37 ± 0.314	0.065
Crude protein	18.09 ± 0.217	19.10 ± 0.303	0.030
Ether extract	7.06 ± 0.905	7.01 ± 1.204	0.928
Ash	2.08 ± 0.203	2.09 ± 0.301	0.978

Note: IW, initial weight; FW, final weight; WGR, weight gain rate; SGR, specific growth rate; SR, survival rate; FI, feed intake; FCR, feed conversion ratio; HSI, hepatopancreas index. The above data were expressed as means ± SEMs.

## Data Availability

The raw dataset for 16SrDNA sequencing has been deposited in the BioSample database (Submission ID: SUB14521244; Bio Sample accessions: SAMN41784796, SAMN41784797, SAMN41784798, SAMN41784799, SAMN41784800, SAMN41784801).
